# The PolyVent educational platform: An open mechanical ventilation platform for research and education

**DOI:** 10.1016/j.ohx.2024.e00615

**Published:** 2025-01-07

**Authors:** Robert L. Read, Nathaniel Bechard, Victor Suturin, Antal Zuiderwijk, Michelle Mellenthin

**Affiliations:** a Public Invention, 1709 Norris Dr. Austin, TX, 78704, United States of America; b Concordia University, 1455 Blvd. De Maisonneuve Ouest, Montreal, QC, H3G 1M8, Canada; c Aliform, Oxfordlaan 55, 6229 EV Maastricht, Netherlands; d Public Invention, De Boerstraat 70, 2685 RG Poeldijk, Netherlands; e Colorado Mesa University, 1100 North Ave, Grand Junction, CO, 81501, United States of America

**Keywords:** Respiration, Ventilation, Pulmonology, Ventilation modes, Patient-ventilator asynchrony (PVA), Pressure-controlled ventilation (PCV)

## Abstract

The PolyVent is an open source mechanical ventilator meant for research and education. It prioritizes openness, modularity, repairability, and modifiability. An ESP32 microcontroller controls a proportional valve which precisely modulates pressure and flow from a mixing chamber into the airway. This chamber is fed with pressurized oxygen and medical air. Solenoid valves control both gas mixing and patient inflation. The PolyVent is controllable through a command-line interface over the serial port, a convenient point of access for researchers and instructors. The VentMon, a separate IoT-enabled spirometer, provides convenient instrumentation for classroom teaching and geodistributed research teams. A “cake-dome” design allows the PolyVent to operate with or without its transparent cover in place, for easy troubleshooting and instruction. An open footprint optimizes engineering change rather than compactness. The electronics are packaged into cards on a standardized backplane, allowing one to extend functionality through the addition of new cards. The VentOS open source software that drives the machine makes it a universal and modifiable research software platform. It is intended to be the medical gas production heart of an open source human respiration research and education ecosystem, and aims to be the starting point for open source medical ventilator designs.

## Graphical abstract

**Figure:**
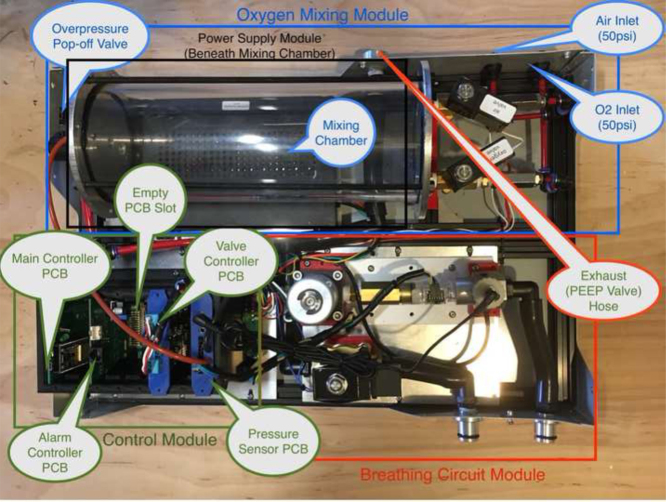

**Specifications table**

**Table:** 

**Hardware name**	PolyVent Educational Platform

**Subject area**	Medical (Pulmonological)

**Hardware type**	Pulmonological research and simulation

**Closest commercial analog**	The closest analog may be the JPL VITAL ventilator [1].

**Open source license**	Hardware: CERN OHL-S (v2) Software: GNU Affero General Public License Documentation: CC0

**Cost of hardware**	The material cost is approximately US$2000. The labor cost is likely significant if outsourced, possibly US$2500.

**Source file repository**	DOI: http://dx.doi.org/10.5281/zenodo.14425536, link: https://zenodo.org/records/14425536

**OSHWA certification UID** *(OPTIONAL)*	US002608

## 1 Hardware in context

The PolyVent is a research and educational mechanical ventilator that shares similar functionality with medical ventilators and CPAP machines, which are currently not intended for use on human patients. Medical ventilators rhythmically produce breaths of tightly controlled pressure, duration, and frequency consisting of an inhalation under positive pressure, and a free exhalation at ambient pressure, or slightly higher. Sometimes exhalations are at slightly above ambient pressure to create positive, end-expiratory pressure (PEEP). A PEEP of 5 cmH_2_O pressure is typically used in most ventilation regimes to prevent potential collapse of airways and alveoli .

Most ventilators use a proprietary design, parts, and closed software. During the COVID-19 pandemic, a large number of emergency ventilators were designed [2], [3]. Some of them were, in spirit, open source. However, few of them actually openly provided full designs without some sort of registration or permission. Many of these were “bag squeezers” which mechanically squeezed valve bag masks. We do not discuss these models further in this paper because they require high maintenance and were aimed primarily at mitigating an expected shortage of ventilators.

Other ventilators use bellows or turbines, which potentially allow them to be stand-alone devices. However, another ventilator design separated the pressurization of clean medical air and therapeutic oxygen (O_2_). The PolyVent team chose this approach because pressurized gas stores a great deal of energy. If the electrical power fails, the ventilator can operate for a long time on a relatively small battery backup. Modern clinics are designed to provide medical air and oxygen at about 50 psi from the wall. Fully open-source ventilators using this design include:

• the SmithVent [4], [5],
• “The People’s Vent” [6],
• the OperationAIR Ventilator [7].

A large number of ventilators are partially open source, but require permission of some kind to even see the design and code. This is the case for devices such as the JPL VITAL ventilator made by NASA [1], for example. Other claimants [8] do not have clear links to open source repositories. At least one inexpensive, open-source unnamed ventilator based on the turbine approach is fully defined in the supplemental material in the publication [9].

The PolyVent could be the basis of derived designs for medical ventilators developed with open-source, “frugal” design, which has been described as a design with only core functionality [10] and without rarely used features. At present, the PolyVent is not intended for medical purposes. However, as a research instrument, it uses a highly modular approach, attempting to create a gas production heart of an “ecosystem” of cooperating and interchangable components that communicate via open standards [11], [12].

Any ventilation of humans or animals would ideally use very clean “medical air”. For most disease conditions, enriching the air with therapeutic oxygen is an absolute requirement. Every ventilator must therefore consider the input gas. The PolyVent controls fractional oxygen percentage (FiO_2_) by accepting separate pressurized air and pressurized O_2_ inputs, an approach used by the award-winning SmithVent [5]. This system has the advantage of being tolerant of power failures, which are common in low and middle income countries (LMICs). The ventilator requires relatively little power to control the valves, and a battery backup would be long-lasting. Pressurized tanks of O_2_ and air remain pressurized for a long time without requiring electricity to increase the pressure.

## 2 Hardware description

The PolyVent is similar to other invasive ventilators, non-invasive ventilators and CPAP machines in terms of its medical gas delivery capabilities. However, the philosophy of open extensibility guides its design. The PolyVent is not an endpoint product, but a platform for development and research. Thus, it offers several unique features in order to be as repairable and extensible as possible. These include:

• a system of “Half Eurocard” IEEE 1101.10 standard [13] pluggable printed circuit board assemblies by which new electronic functionality can be added without modification to the existing design,
• a “cake dome” design that supports classroom instruction and easy demonstration,
• a spacious footprint that allows working room for easy repair and modification,
• the VentOS universal ventilator software [14],
• the use of a separate spirometer [15],
• the use of open data standards for both control and data output.

The PolyVent is extendable with new electronic features and is built on the Half Eurocard PCB mechanical standard. The main microcontroller (see Fig. 5) is itself just one of several cards installed into the backplane, as shown in Figs. 28, 33, and 36. The valve control card, which provides 24 V power to operate valves and the sensor interface cards, are similarly Half Eurocard PCBs slotted into the backplane. The card expansion system provides great flexibility in extending the functionality, just as it did for personal computers in the 1990s. By using well-established mechanical format for pluggable cards, which provides 24 V power and makes accessible the large number of GPIO pins of the ESP32 main controller accessible, new features can be added. For example, an alarm system using the General Purpose Alarm Device (GPAD) was added via the design of an interfacing card [16]. Future expansions could include a heater, humidifier, or a drug nebulizer, which one could control through GPIO pins and drive with 24 V power.

The PolyVent has its own internal flow and pressure sensors which directly control the ventilation regime. However, as part of the PolyVent ecosystem, we implement a separate spirometer, the VentMon [15], an IoT enabled instrument which supports independent testing and analysis of the PolyVent. The VentMon has been the primary window into the PolyVent’s performance during classroom instruction.

Repairability in low-resource settings was a major goal of the PolyVent, recently identified as a major global need [17], [18]. Humanitarian advocacy, such as the “Right To Repair” movement [19], [20], argues for legal repairability, which presupposes technical repairability.

The PolyVent has a modular physical layout with a spacious footprint surmounted by a “cake dome” style cover that a user can lift on and off while the ventilator operates. The cover is transparent to match our figurative design transparency. These features make the PolyVent easy to understand, repair, and test, though they make it less compact than ventilators designed for high-resource settings.

The PolyVent is ideal for classroom instruction, since it can be safely operated with the cover off and observed by approximately eight students simultaneously. Minor modifications, such as temporarily, intentionally installing a leaky hose or obstructing an airway, can be easily presented as troubleshooting exercises. In combination with a test lung, the VentMon or other spirometer, and VentDisplay software, the PolyVent can demonstrate principles of:

• pulmonology,
• physics concepts of volume, flow, and pressure,
• mechatronics,
• embedded electronics, and
• global health.

Research that requires a change in ventilation under algorithmic control not provided by existing ventilators, such as:

• new ventilation modes [21],
• new approaches to addressing patient-ventilator asynchrony [22], or
• new integration of sensor input such as thoracic pressure [23]

are possible by quickly reprogramming the PolyVent. These would be very difficult to accomplish with a closed ventilator.

US non-profit Helpful Engineering independently began the VentOS software project in 2020 as a universal control software for ventilators. It relies on the principle of a Hardware Abstraction Layer (HAL) implemented by hardware-specific drivers to attempt to be universal. Although no other team has yet attempted to use VentOS in this way, it provides flexibility to the PolyVent. For example, it is driven by the Public Invention Respiratory Control Standard (PIRCS), which is meant to be a universal interface for clinical respiration commands and makes the user interface independent and modular. VentOS is controlled by commands typed into the serial port in the PIRCS standard format.

## 3 Design files summary

**Table:** 

Design filename	File type	Open source license	Location of the file
Education PolyVent System CAD	Central Design, available in .f3d and .step formats	CERN Open Hardware Licence Version 2 - Strongly Reciprocal	https://zenodo.org/records/14425536/preview/PolyVent.zip?include_deleted=0#tree_item6

Control Module	Module, available in .f3d and .step formats	CERN Open Hardware Licence Version 2 - Strongly Reciprocal	(Boards) https://zenodo.org/records/14425536/preview/PolyVent.zip?include_deleted=0#tree_item24 (Wiki) https://zenodo.org/records/14425536/preview/PolyVent.zip?include_deleted=0#tree_item182

ESP32 card V2.2	Component, available in .f3d and .step CAD formats and in Eagle .sch and .brd board files	CERN Open Hardware Licence Version 2 - Strongly Reciprocal	(CAD) https://zenodo.org/records/14425536/preview/PolyVent.zip?include_deleted=0#tree_item41 (Board) https://zenodo.org/records/14425536/preview/PolyVent.zip?include_deleted=0#tree_item37

Sensing card V2.2	Component, available in .f3d and .step CAD formats and in Eagle .sch and .brd board files	CERN Open Hardware Licence Version 2 - Strongly Reciprocal	(CAD) https://zenodo.org/records/14425536/preview/PolyVent.zip?include_deleted=0#tree_item66 (Board) https://zenodo.org/records/14425536/preview/PolyVent.zip?include_deleted=0#tree_item62

Valves Card V2.1	Component, available in .f3d and .step CAD formats and in Eagle .sch and .brd board files	CERN Open Hardware Licence Version 2 - Strongly Reciprocal	(CAD) https://zenodo.org/records/14425536/preview/PolyVent.zip?include_deleted=0#tree_item66 (Board) https://zenodo.org/records/14425536/preview/PolyVent.zip?include_deleted=0#tree_item71

Prototype board	Component, available in Eagle .sch and .brd board files. This board adds extra functionality to the PolyVent	CERN Open Hardware Licence Version 2 - Strongly Reciprocal	(Board) https://zenodo.org/records/14425536/preview/PolyVent.zip?include_deleted=0#tree_item57

Backplane	Component, available in Eagle .sch and .brd board files	CERN Open Hardware Licence Version 2 - Strongly Reciprocal	(Board) https://zenodo.org/records/14425536/preview/PolyVent.zip?include_deleted=0#tree_item24

guide_box_base	Component, .stl file, editable file available in the Central Design	CERN Open Hardware Licence Version 2 - Strongly Reciprocal	https://zenodo.org/records/14425536/preview/PolyVent.zip?include_deleted=0#tree_item116

guide_box	Component, .stl file, editable file available in the Central Design	CERN Open Hardware Licence Version 2 - Strongly Reciprocal	https://zenodo.org/records/14425536/preview/PolyVent.zip?include_deleted=0#tree_item116

PSU Enclosure Lid	Component, .stl file, editable file available in the Central Design	CERN Open Hardware Licence Version 2 - Strongly Reciprocal	https://zenodo.org/records/14425536/preview/PolyVent.zip?include_deleted=0#tree_item123

PSU Enclosure Base	Component, available in .step, .pdf, and .dxf file formats	CERN Open Hardware Licence Version 2 - Strongly Reciprocal	https://zenodo.org/records/14425536/preview/PolyVent.zip?include_deleted=0#tree_item119

Gas Mixing Module	Module, available in .f3d and .step formats	CERN Open Hardware Licence Version 2 - Strongly Reciprocal	https://zenodo.org/records/14425536/preview/PolyVent.zip?include_deleted=0#tree_item381 https://zenodo.org/records/14425536/preview/PolyVent.zip?include_deleted=0#tree_item379

Input Endplate	Component, available in .step, .pdf, and .dxf file formats	CERN Open Hardware Licence Version 2 - Strongly Reciprocal	https://zenodo.org/records/14425536/preview/PolyVent.zip?include_deleted=0#tree_item385

Output and Sensor Endplate	Component, available in .step, .pdf, and .dxf file formats	CERN Open Hardware Licence Version 2 - Strongly Reciprocal	https://zenodo.org/records/14425536/preview/PolyVent.zip?include_deleted=0#tree_item389

TPU Tank mount	Component, available in .step, .pdf, and .dxf file formats	CERN Open Hardware Licence Version 2 - Strongly Reciprocal	https://zenodo.org/records/14425536/preview/PolyVent.zip?include_deleted=0#tree_item393

Gas Drive Module	Module, available in .f3d and .step formats	CERN Open Hardware Licence Version 2 - Strongly Reciprocal	https://zenodo.org/records/14425536/preview/PolyVent.zip?include_deleted=0#tree_item340

Flow Conditioner Grid	Component, .stl file, editable file available in the Central Design	CERN Open Hardware Licence Version 2 - Strongly Reciprocal	https://zenodo.org/records/14425536/preview/PolyVent.zip?include_deleted=0#tree_item123

Flow Conditioner Housing	Component, available in .step, .pdf, and .dxf file formats	CERN Open Hardware Licence Version 2 - Strongly Reciprocal	https://zenodo.org/records/14425536/preview/PolyVent.zip?include_deleted=0#tree_item351

Gas Drive Base Plate	Component, available in .step, .pdf, and .dxf file formats	CERN Open Hardware Licence Version 2 - Strongly Reciprocal	https://zenodo.org/records/14425536/preview/PolyVent.zip?include_deleted=0#tree_item355

Manifold Damper	Component, .stl file, editable file available in the Central Designs	CERN Open Hardware Licence Version 2 - Strongly Reciprocal	https://zenodo.org/records/14425536/preview/PolyVent.zip?include_deleted=0#tree_item359

Proportional Valve Damper	Component, .stl file, editable file available in the Central Design	CERN Open Hardware Licence Version 2 - Strongly Reciprocal	https://zenodo.org/records/14425536/preview/PolyVent.zip?include_deleted=0#tree_item363

Sensing Manifold	Component, available in .step, .pdf, and .dxf file formats	CERN Open Hardware Licence Version 2 - Strongly Reciprocal	https://zenodo.org/records/14425536/preview/PolyVent.zip?include_deleted=0#tree_item365

PIV Module	Module, available in .f3d and .step formats	CERN Open Hardware Licence Version 2 - Strongly Reciprocal	https://zenodo.org/records/14425536/preview/PolyVent.zip?include_deleted=0#tree_item343

PIV Damper	Component, .stl file, editable file available in the Central Design	CERN Open Hardware Licence Version 2 - Strongly Reciprocal	https://zenodo.org/records/14425536/preview/PolyVent.zip?include_deleted=0#tree_item361

Frame and Cover	Module, available in .f3d and .step formats	CERN Open Hardware Licence Version 2 - Strongly Reciprocal	https://zenodo.org/records/14425536/preview/PolyVent.zip?include_deleted=0#tree_item263

Control Module Baseplate	Component, available in .step, .pdf, and .dxf file formats	CERN Open Hardware Licence Version 2 - Strongly Reciprocal	https://zenodo.org/records/14425536/preview/PolyVent.zip?include_deleted=0#tree_item302

PIV and Gas Drive Baseplate	Component, available in .step, .pdf, and .dxf file formats	CERN Open Hardware Licence Version 2 - Strongly Reciprocal	https://zenodo.org/records/14425536/preview/PolyVent.zip?include_deleted=0#tree_item306

Tub	Component, available in .step and .dxf file formats	CERN Open Hardware Licence Version 2 - Strongly Reciprocal	https://zenodo.org/records/14425536/preview/PolyVent.zip?include_deleted=0#tree_item318

Base Frame	Component, available in .step, .pdf, and .dxf file formats	CERN Open Hardware Licence Version 2 - Strongly Reciprocal	https://zenodo.org/records/14425536/preview/PolyVent.zip?include_deleted=0#tree_item295

Mounting Bar	Component	CERN Open Hardware Licence Version 2 - Strongly Reciprocal	The Mounting bar is simply a 146 mm long piece of 2020 aluminum

Acrylic Lid Left Side	Component, available in .step and .f3d formats	CERN Open Hardware Licence Version 2 - Strongly Reciprocal	https://zenodo.org/records/14425536/preview/PolyVent.zip?include_deleted=0#tree_item282

Acrylic Lid Right Side	Component, available in .step and .f3d formats	CERN Open Hardware Licence Version 2 - Strongly Reciprocal	https://zenodo.org/records/14425536/preview/PolyVent.zip?include_deleted=0#tree_item285

Acrylic Lid Top	Component, available in .step, .f3d, and .dxf formats	CERN Open Hardware Licence Version 2 - Strongly Reciprocal	https://zenodo.org/records/14425536/preview/PolyVent.zip?include_deleted=0#tree_item288

Acrylic Lid Bending Jig	Jig, available in .step and .f3d formats	CERN Open Hardware Licence Version 2 - Strongly Reciprocal	https://zenodo.org/records/14425536/preview/PolyVent.zip?include_deleted=0#tree_item299

Plate Mount 22 mm Hose Coupler	Component, available in .step, .pdf, and .dxf file formats	CERN Open Hardware Licence Version 2 - Strongly Reciprocal	https://zenodo.org/records/14425536/preview/PolyVent.zip?include_deleted=0#tree_item310

Plate Mount 22 mm Hose Coupler Female	Component, available in .step, .pdf, and .dxf file formats	CERN Open Hardware Licence Version 2 - Strongly Reciprocal	https://zenodo.org/records/14425536/preview/PolyVent.zip?include_deleted=0#tree_item314

This is a rather large system that has numerous design files. These include:

• printed circuit board assembly designs (generally Eagle CAD source files),
• machinable parts, presented as drafting files (.pdfs), .dxf files and .step file CNC production files.
• 3D printable files presented as Fusion 3D (.f3d) source files and .stl files or .step files.

The 36 directories mentioned in the tables above are summarized below:

1. Education PolyVent System CAD — A general model of the entire machine, usable for education and understanding the design. We used this file during the initial design process.
2. Control Module — This is a European half-card based, 3D printed control system. It is a physical design that allows for card-based expansion.
3. ESP32 card V2.2 — The most important of our control printed circuit assemblies, this is the main microcontroller (an ESP32) which runs the VentOS software and is the heart of the control module. The pins of the ESP32 are tied via the backplane to other cards.
4. Sensing card V2.2 — The sensing card holds the pressure sensor (connected by a small tube to the airway). This can expand to other sensors.
5. Valves Card V2.1 — The valves card contains 24 V transistors for the control of the solenoid valves. This has a distinct, small microcontroller which communicates with the main ESP32 and allows the timing issues associated with motor, valve, and solenoid control to separate from the main controller’s timing.
6. Prototype board — A prototype board or card which allows components to be easily added with through-hole soldering. It also represents an initial design point for new cards. This is a significant means of explaining functionality.
7. Backplane — The backplane Eagle CAD assembly is installed at the bottom of the control module, which ties together the expansions cards in terms of signals (on GPIO pins) and provides 24 V power and ground.
8. guide_box_base — Defines the 3D printable base into which the expansion card assemblies can be plugged.
9. guide_box — This is the 3D printable box that “guides” the expansion and control printed circuit boards via slots. These slots makes sure they plug properly into the guide_box_base and provides mechanical support for them.
10. PSU Enclosure Lid — This is a mechanical enclosure for the Power Supply Unit. The main purpose of this is to prevent accidental electrical shock during education, but it also provides mechanical protection. This contains the power supply that converts 110VAC power to DC 5 V, 12 V and 24 V power.
11. PSU Enclosure Base — This includes the drafting and DXF files for the metal Power Supply Enclosure Base. The power supplies are screwed into this piece of metal, which is in turn screwed into the 20/20 extrusion rails mounted to the ventilator base (the “tub”). These are mounted with M3 and M4 machine screws.
12. Gas Mixing Module — Shows the F3Z and .step files for the base of the gas mixing module. This module is mounted to the 2020 extrusion.
13. Input Endplate — The CNC-able and drafting design of the input endplate. In additional to the mounting holes, there are holes for the threaded rods that keep the Endplates pressed against the acrylic missing tube. It also describes port holes for the solenoid input valves for air and O_2_ input.
14. Output and Sensor Endplate — The Output and Sensor Endplate is similar to the Input in form and mounting, but has ports for a pressure sensor, the over-pressure safety release valve, and the output port itself.
15. TPU Tank mount — The Thermoplastic Polyurethane tank mount is a 3D printable mount which is useful for absorbing sound and vibration. By using soft TPU as a material, we significantly reduced vibration and noise.
16. Gas Drive Module — This is a design of the for planning and design of the buildable mounts for all of the components of the Gas Drive Module. These include the proportional valve, a filter, the flow sensor, the pressure sensor, the FiO_2_ sensor, and a right angle bend.
17. Flow Conditioner Grid — This is a 3D printable honeycomb which will laminarize the flow coming out of the proportional valve, which may otherwise be turbulent. Laminar flow is required by the flow and pressure sensors.
18. Flow Conditioner Housing — This is a hexagonal machinable part that can be machined from a piece of hexagonal aluminum. It holds the Flow Conditioner Grid in the airway.
19. Gas Drive Base Plate — This a .step and .f3z file for the metal plate on which the Gas Drive Module is mounted. The various component mounts are mounted here, and the Base Plate itself is mounted on the 2020 extrusion rails, allowing the entire module to be redesigned, modified, or swapped out.
20. Manifold Damper — This is a TPU damper that limits vibration in the sensing manifold as part of the Gas Drive Module.
21. Proportional Valve Damper — This is a TPU damper specifically for the Proportional Valve, which is probably the loudest part in the system.
22. Sensing Manifold — Shows the machinable manifold that holds the pressure sensor and FiO_2_ sensor in place. It is machinable with a lathe out of hexagonal aluminum.
23. PIV Module — The general model of the Patient Inflating Valve Module.
24. PIV Damper — A TPU vibration-damping bracket that holds the Patient Inflating valve (PIV). The PIV valve is one of the heaviest and loudest components within the system.
25. Frame and Cover — This contains the flat (pre-forming) pattern for the acrylic cover.
26. Control Module Baseplate — Shows the metal baseplate upon which the control guide box is mounted.
27. PIV and Gas Drive Baseplate — This is drafting of the 0.125in aluminum baseplate with mounting holes for the PIV and Gas Drive mounting components.
28. Tub — Includes .dxf and .step files for the metal “tub” which holds all modules via 2020 extrusion.
29. Base Frame — A drafting of the cuts needed on the 2020 extruded aluminum and the placement of tapped M5 holes.
30. Mounting Bar — There is no file for this because it is simply a 146 mm long piece of 220 aluminum.
31. Acrylic Lid Left Side — This is the flat, 2D design of the acrylic left side.
32. Acrylic Lid Right Side — This is the flat, 2D design of the acrylic right side.
33. Acrylic Lid Top — This is the flat, 2D design of the acrylic lid top.
34. Acrylic Lid Bending Jig — Includes the design of a plywood jig for bending the gentle curves in the acrylic lid top (cover) with a heat gun. By operating slowly with the help of this jig, it is possible to form an attractive acrylic cover without bubbling the acrylic.
35. Plate Mount 22 mm Hose Coupler — Shows a design of the machinable hose coupler which is mounted in the frame (tub) on both the inside and the outside. Typically machined from aluminum, this is a standard 22 mm taper-fit connector for a breathing circuit. It is bolted through the tub.
36. Plate Mount 22 mm Hose Coupler Female — Describes the female (interior to the but) hose coupler with a 22 mm female orifice for connection of the short airway hoses inside the ventilator.

## 4 Bill of materials summary

**Table:** 

Designator	Component	Number	Cost per unit - USD	Total cost - USD	Source of materials	Material type
Control Module	see BOM file on Zenodo	1	587.18	587.18	https://zenodo.org/records/14425536/preview/PolyVent.zip?include_deleted=0#tree_item22	Semiconductor

Gas Mixing Module	see BOM file on Zenodo	1	243.52	243.52	https://zenodo.org/records/14425536/preview/PolyVent.zip?include_deleted=0#tree_item336	Metal and Polymer

PIV and Gas Drive Module	see BOM file on Zenodo	1	858.09	858.09	https://zenodo.org/records/14425536/preview/PolyVent.zip?include_deleted=0#tree_item336	Metal, Semiconductor, and Polymer

Frame and Cover Module	see BOM file on Zenodo	1	223.28	223.28	https://zenodo.org/records/14425536/preview/PolyVent.zip?include_deleted=0#tree_item261	Metal and Polymer

## 5 Build instructions

**Preparatory Notes**

This assembly document provides tips and complications to watch out for during the build process. There are details missing in the instructions, and the CAD should be used as a reference for these gaps. For example, these instructions assume that you have already 3D printed the enclosure for the control module. Additionally, the machining and printing instructions are not included here. This document assumes you already have all the parts made.

**Add silicone to the endcaps**

Make sure to use 100% silicone sealant. Black silicone works better since you will be able to see if it has sealed to the tube later.

**Fig. 1:**
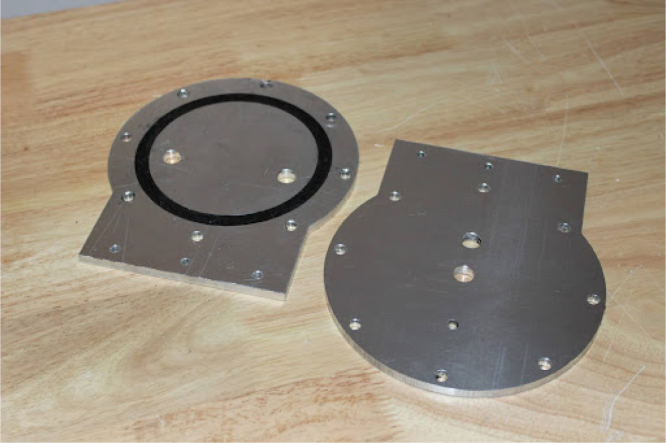
End caps.

• Squeeze silicone into the spot for the gasket and make sure it fills the space fully.
• Make sure that all other surfaces are covered with masking tape. Leave to cure for 24–48 h.
• The final product will look like Fig. 1.
• Set the polycarbonate for the plastic case to dry. Depending on the process and temperature used for drying this can take hours or days.

**Make the base frame out of 2020 T-slot extruded rails**

Use the design file for it: Base Frame Manufacturing layout.

**Fig. 2:**
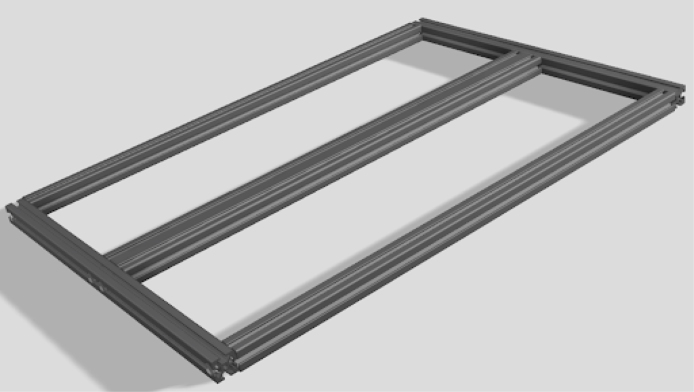
Frame.

**Fig. 3:**
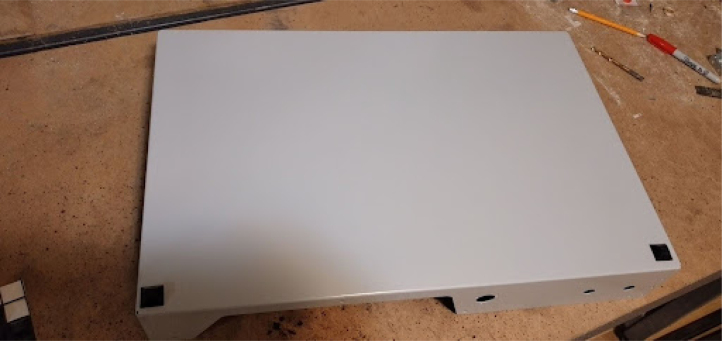
Base tub with rubber feet.

• Use a miter saw or hacksaw to cut the 2020 and drill it with a drill press. Use a set of thread taps to tap the M6 holes.
• Fasten together with M6 screws. It will look like Fig. 2.

**Mount rubber feet to the base tub**

Shown in Fig. 3. This step prevents scratching.

• Mount the base frame to the tub using 4 M5 flathead screws.
• If the bottom base tub holes are not countersunk already, you will have to do it yourself.

**Install rivets in the tub**

It is important to do this after the 2020 frame is mounted to the base tub, otherwise it may not fit.

**Fig. 4:**
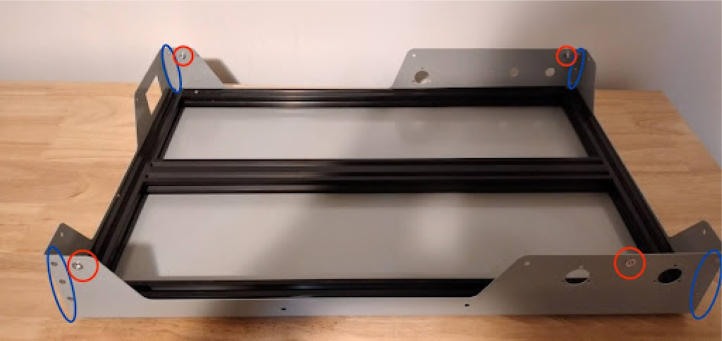
Base with rivets. Nut rivet positions are in red, and double-flush rivet positions are in blue.

• Use double-flush rivets on the corners and nut rivets on the front and back.
• The double-flush rivets will need a countersink if the sheet metal manufacturing house did not already include them. There will be 4 nut rivets and 12 double-flush rivets in total, depicted in Fig. 4. Make sure to get double flush rivets for the right plate thickness.

**Make Control module card and PSU enclosure**

**Fig. 5:**
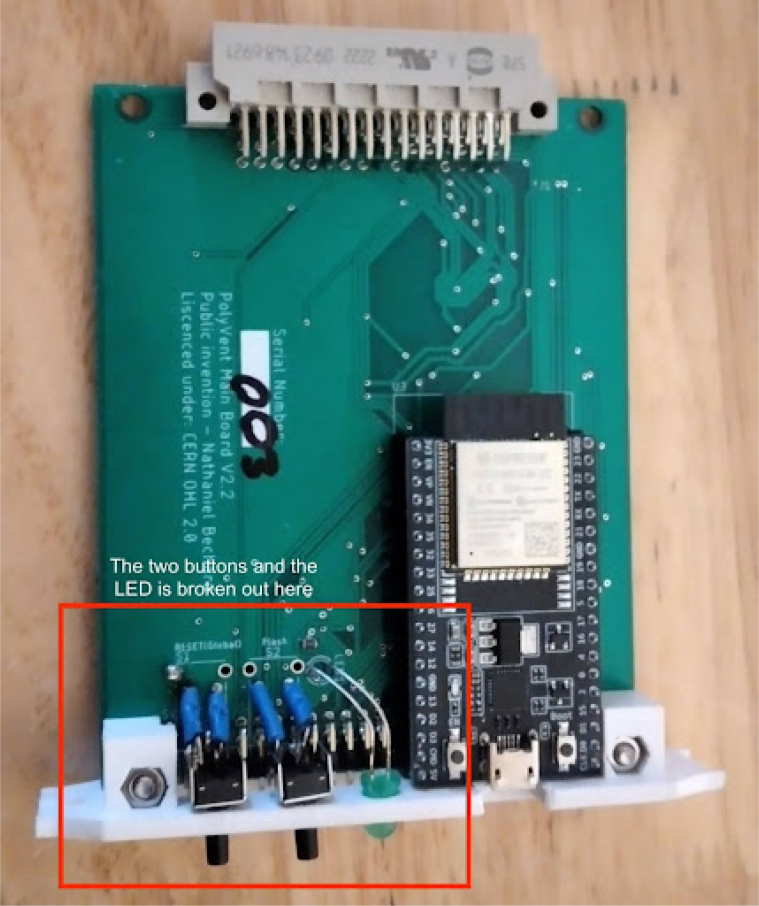
LEDs and buttons on main card.

The sensing card and main card need some modifications that are not obvious from the schematics or CAD. The main card has a 5 mm LED and two buttons broken out and glued to the front panel. It will look like Fig. 5. The recommended 3D print settings for the Control Module can be found here: Print instructions

**Fig. 6:**
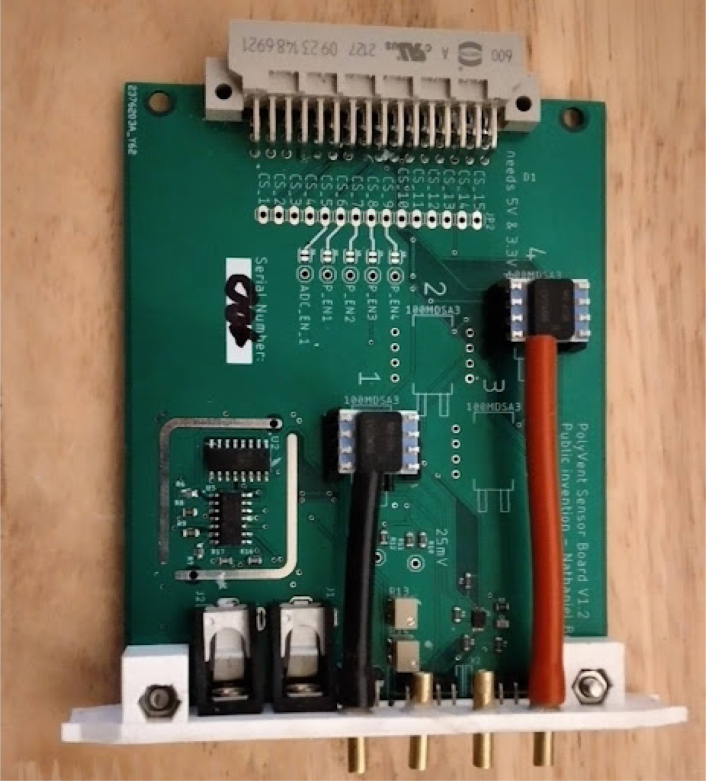
Hoses added to sensing card.

• Glue four brass tubes into the sensing card.
• Then, run some flexible tubing through the card as well.
• The tubes in Fig. 6 are color coded. Maintaining a consistent color-coding is a best practice that we recommend.
• In PolyVent machines, red tubing is used for high pressure and black tubing is used for low pressure.
• Make the tube slightly longer than needed. This precaution keeps the tube in slight compression and helps it stay on the hose barbs, as in Fig. 6.

Otherwise the control module assembly is quite straightforward. Use the control module BOM and CAD to assemble this.

**Make the gas mixing module**

Once the endcaps are dry, the mixing module can be assembled.

**Fig. 7:**
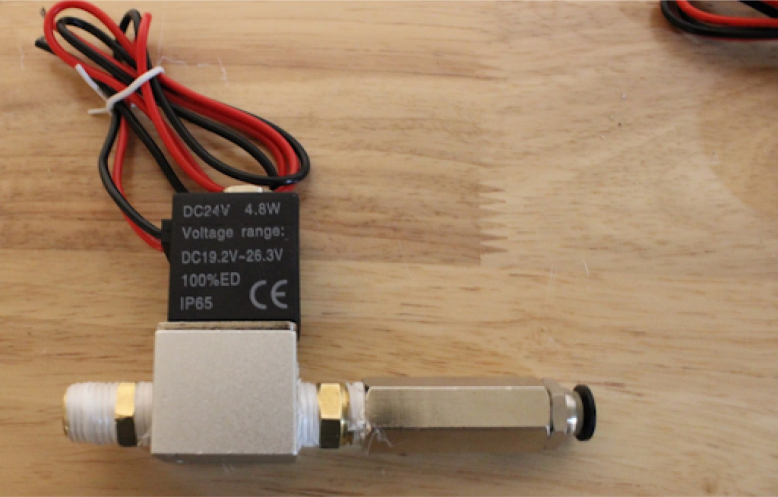
Solenoid pipe fittings.

**Fig. 8:**
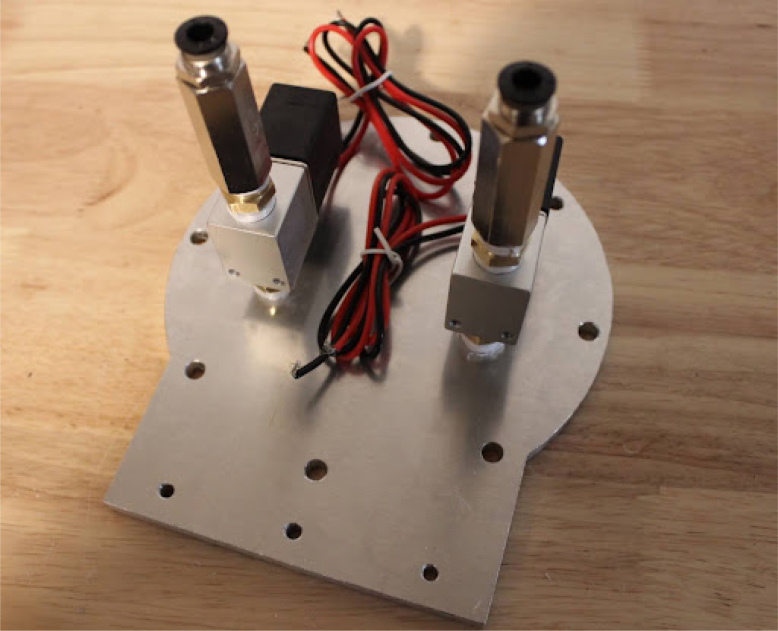
Solenoid valves mounted on endcap.

**Fig. 9:**
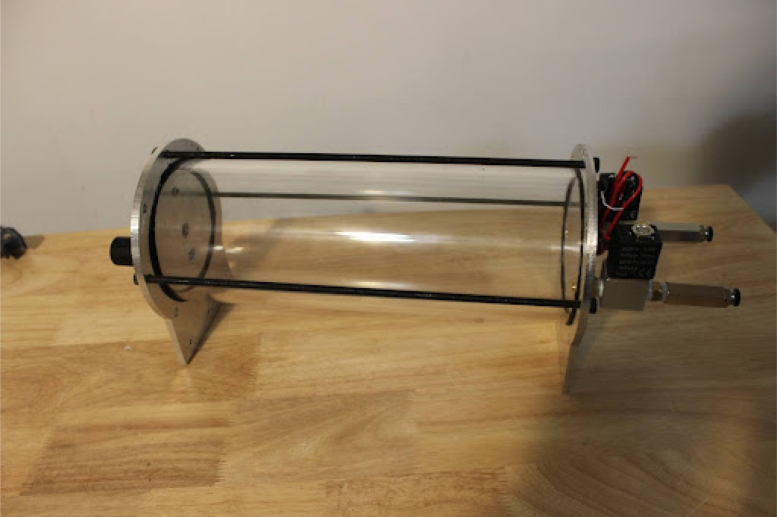
Mixing chamber assembly.

• Make two of these subassemblies. Your valves may differ, but they must be small enough to screw into the input plate without hitting each other. Make sure that the check valve allows flow from right to left (in Fig. 7), from the hexagonal fitting to the valve. You may need a lot of torque to install these pipe fittings, so prepare large wrenches and a vice to hold the valve, as shown in Fig. 7.
• Add the valve assemblies to the input endcap. This may also take a lot of torque. Make sure that your NPT (National Pipe Thread) threads are well formed in the input endcap. The result will look like Fig. 8.
• Install the necessary hardware on the output endcap: the over-pressure valve, pressure sensor hose barb, and output coupler (8 mm right-angle quick-connect fitting). This will be your last chance to clean the acrylic tube and inner endcap surfaces. Sandwich the clear acrylic tube between both plates, making sure that it only touches the silicone gaskets. Tighten the tank bolts until a seal is seen everywhere on both plates.
• Make sure that the over-pressure valve is set to 50PSI (if it is adjustable) before testing the tank by pumping compressed air into it. Raise the pressure in the tank up to 50 PSI slowly using a pressure regulator. If the system fails, it will be due to a leaking gasket. The mixing module will look like Fig. 9 when done.

**Make the gas drive module**

**Fig. 10:**
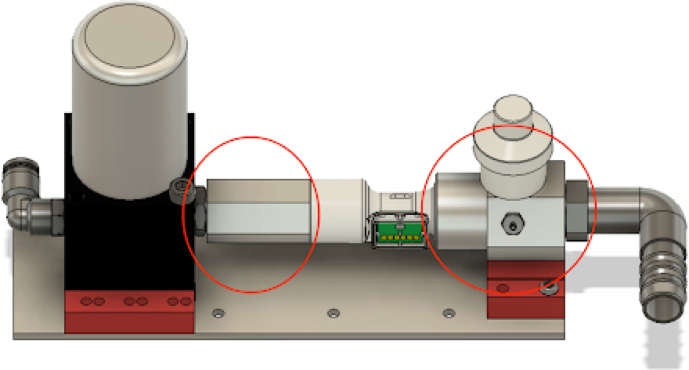
Gas drive module mounting.

Mounting the gas drive module components is quite complicated, as per Fig. 10. The flow stabilizer and manifold circled in Fig. 10 need to be machined so parts can be threaded and inserted perfectly.

**Fig. 11:**
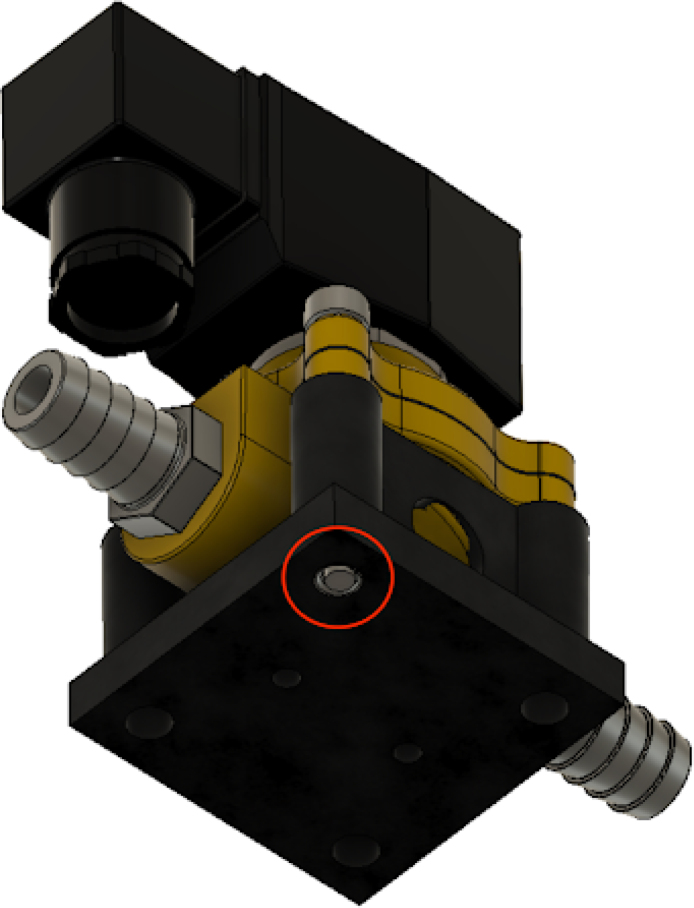
Patient inflating valve assembly.

**Fig. 12:**
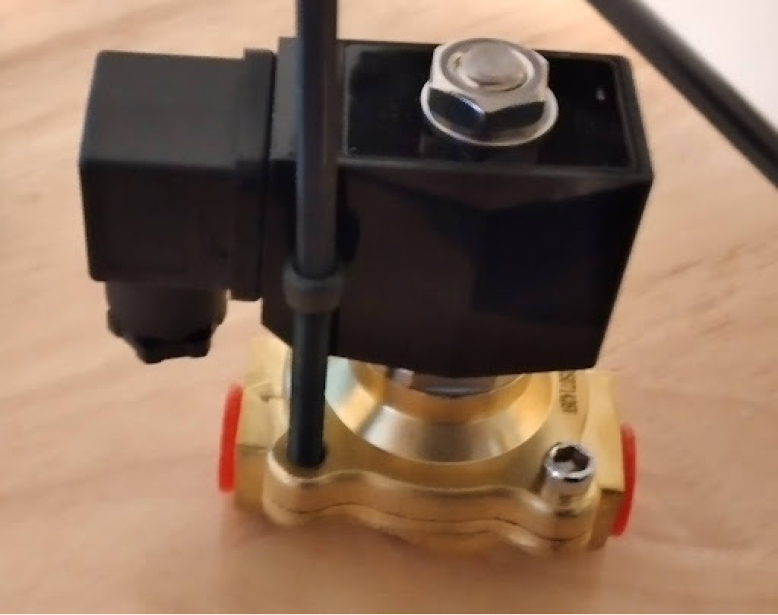
Patient inflating valve mounting bolts.

• The flow stabilizer is made up of two parts: a body, with a flow sensor connector on one side and an NPT thread on the other, and a flow stabilizing grid, which is a small resin printed honeycomb grid which ensures that the flow entering the flow sensor is laminar.
• If these two parts are not properly machined, the dampers (red 3D printed parts) will not align with the holes in the plate.
• The parts need to be threaded together for everything to fit. This will take a lot of torque in the case of the part coming out of the proportional valve.
• The proportional valve needs to be clamped into a vice, and the flow stabilizer needs to be threaded in with a large wrench. See Fig. 11.

**Patient Inflating Valve (PIV) system**

• Add M6 heat set inserts to the bottom of the PIV damper. To keep the valve as quiet as possible, no metal should be touching the base plate. The heat set inserts should not stick out.
• Next, install the hose barbs on the valve and replace the stock M6 bolts with 50 mm M6 bolts, as in Fig. 12.
• Mount the PIV damper to the baseplate before threading in the 50 mm M6 bolts into the inserts. Make sure that the PIV valve has its output pointing left.

**Mount the gas drive module on the gas drive and PIV baseplate**

**Fig. 13:**
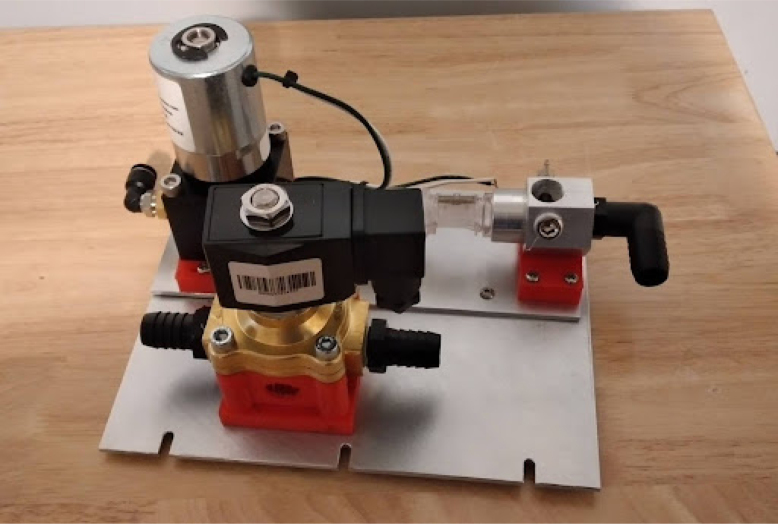
Gas drive module on base plate.

It should look like Fig. 13.

**Fig. 14:**
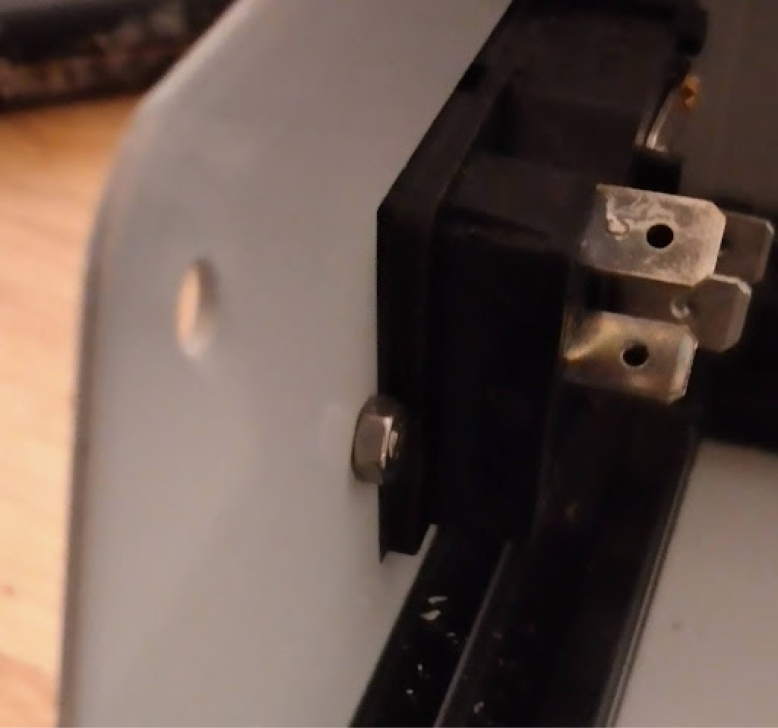
Power block with M3 screws.

**Fig. 15:**
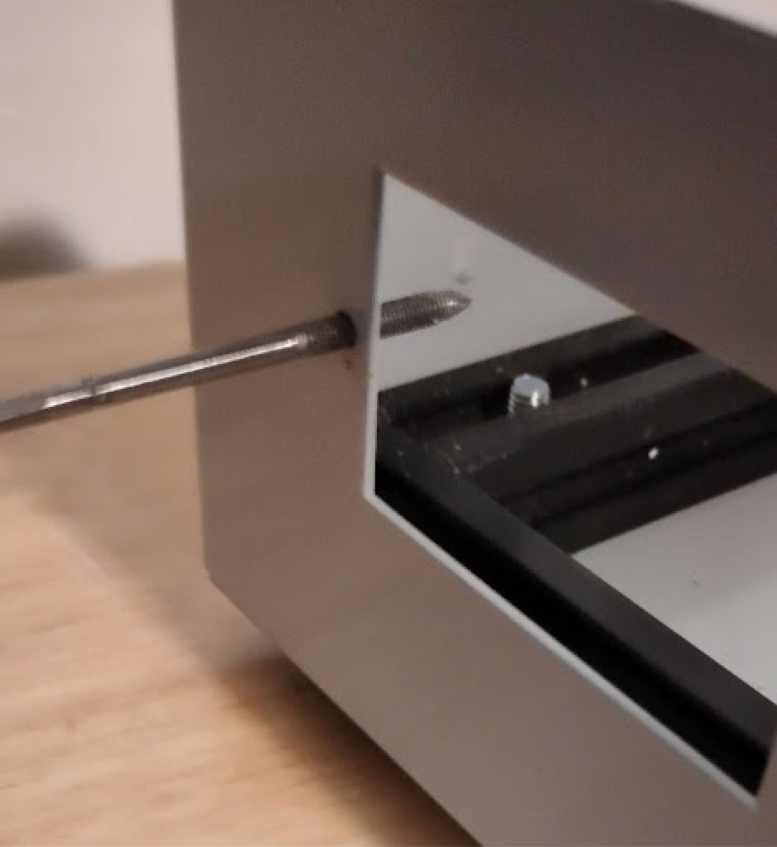
Tapping screw threads.

**Fig. 16:**
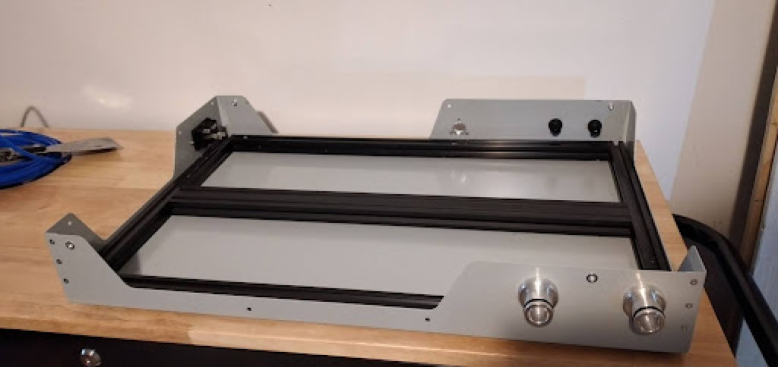
Base with couplers.

**Fig. 17:**
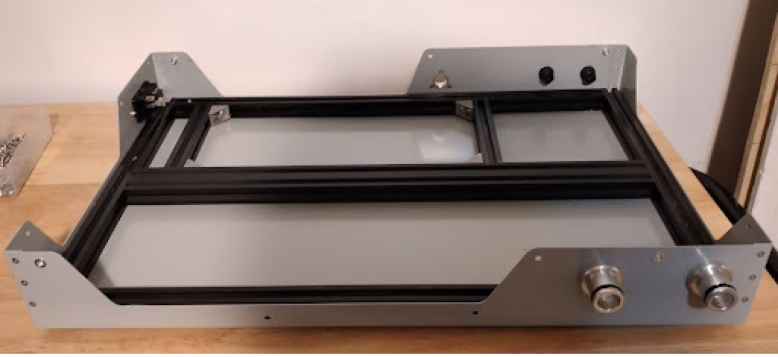
Mixing module 2020 rails added.

**Fig. 18:**
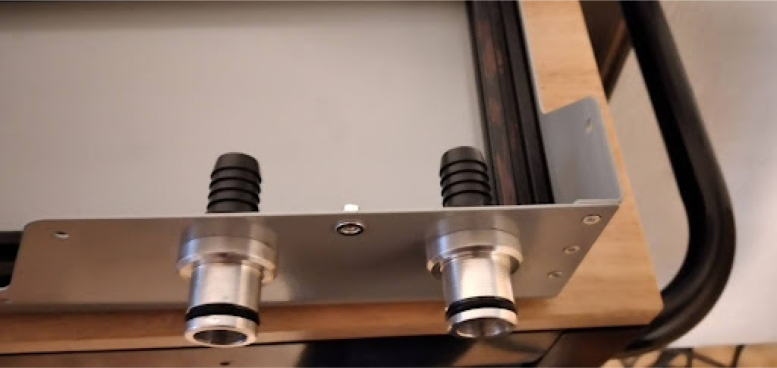
Patient coupler and PEEP hose barbs.

**Fig. 19:**
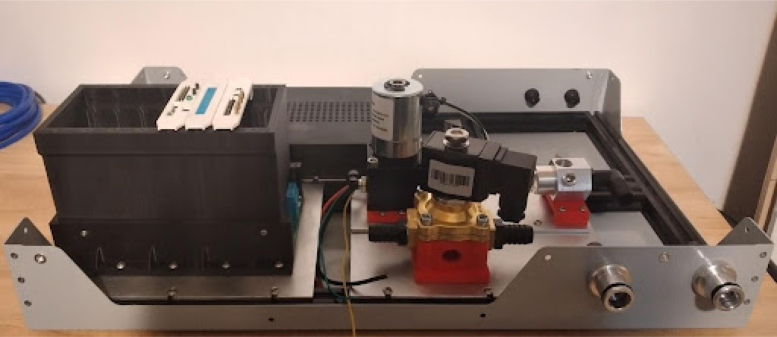
Control module, PIV and gas drive assemblies.

• Install the patient hose couplers, air and oxygen inputs, peep coupler, and power block. The power block needs M3 screws. Ideally the threads for these are tapped into the metal, but if your threads do not hold, nuts can tighten the block down further. See Fig. 14, Fig. 15.
• Use M3 bolts that are 4 mm long to mount the patient couplers and PEEP couplers to the frame. After this step is done, the base will look like Fig. 16.
• Add two 146 mm long 2020 rails for the mixing module. Mount these with T nuts. Mount the corner brackets in the right locations (found in the CAD) then add the rails. It will look like Fig. 17 when installed.
• Thread in the patient coupler and PEEP hose barbs. It will look like Fig. 18 when done.
• Add the control module parts, and the PIV and gas drive assembly. These are mounted on the rails with T nuts as shown in Fig. 19.

**Wiring and tube routing Round 1**

**Fig. 20:**
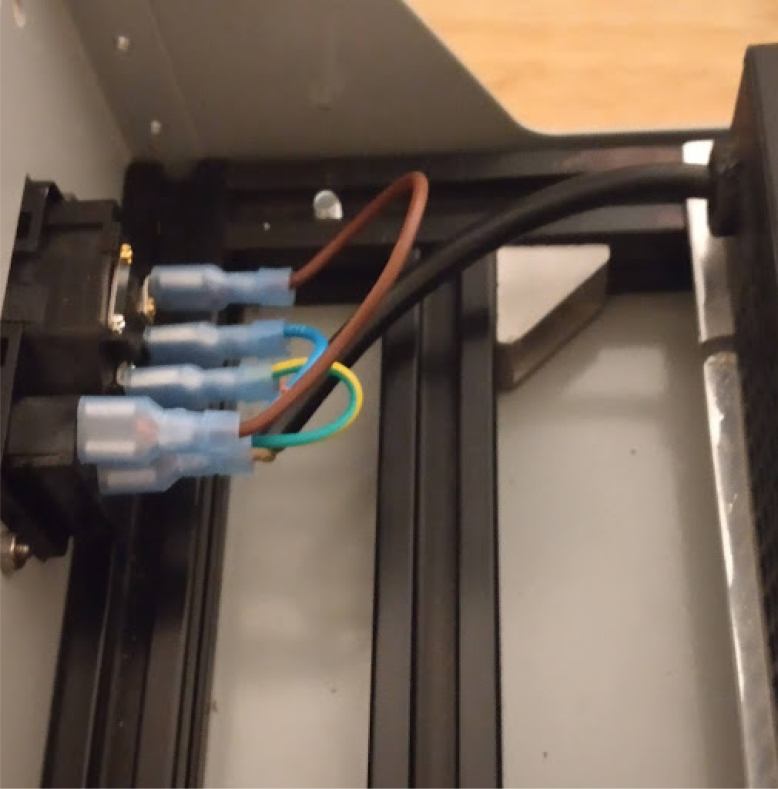
AC wiring.

Some wiring and tube routing is far easier to do before adding the mixing module, so they should be done first. These parts of the wiring and tube routing are: the PEEP line and the AC wiring. Please consult with a professional electrician to complete this step. Fig. 20 shows what the completed AC wiring looks like.

**Fig. 21:**
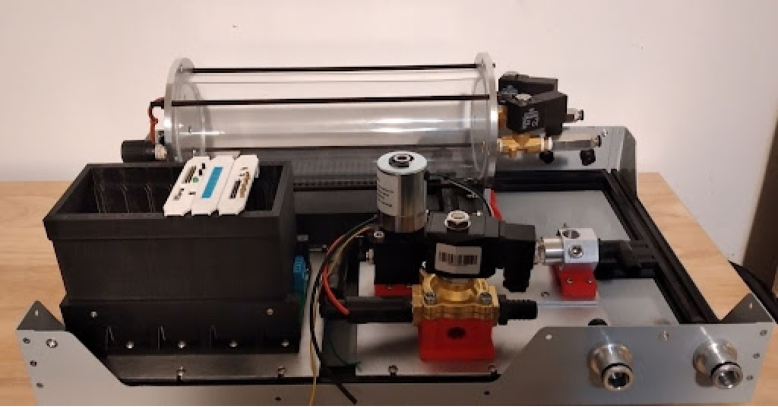
Mixing module mounted.

• Make sure that there is enough wire to go around the mixing module when it gets installed. Your black wire should be a little longer than the one in the image. There is also wiring in the PSU box. This is 110 V AC wiring and has circuit risks.
• The PEEP line is made of 5/8 inch silicone tubing. Use the CAD for reference. Fig. 21 shows this.
• Mount the Mixing module on its rails with T nuts.

**Rout the rest of the tubing**

**Fig. 22:**
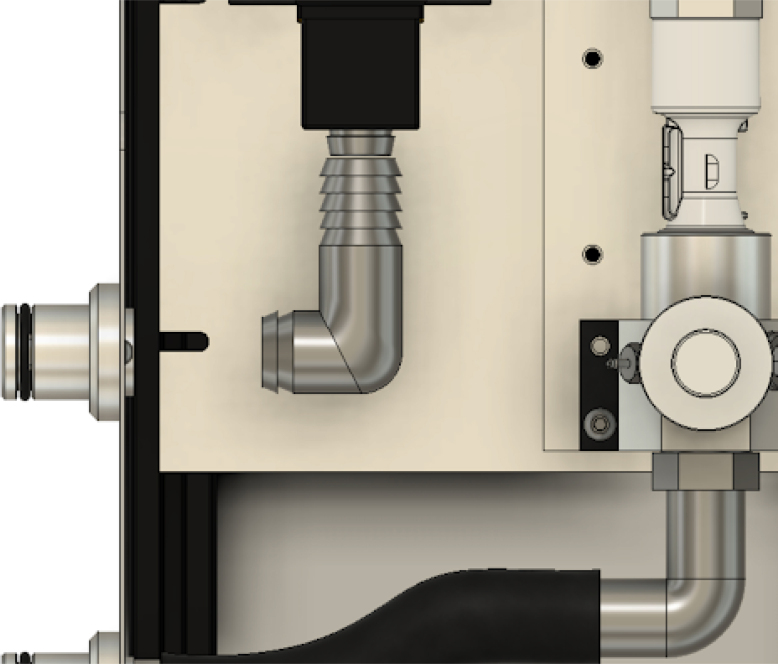
90 degree coupler in the patient.

**Fig. 23:**
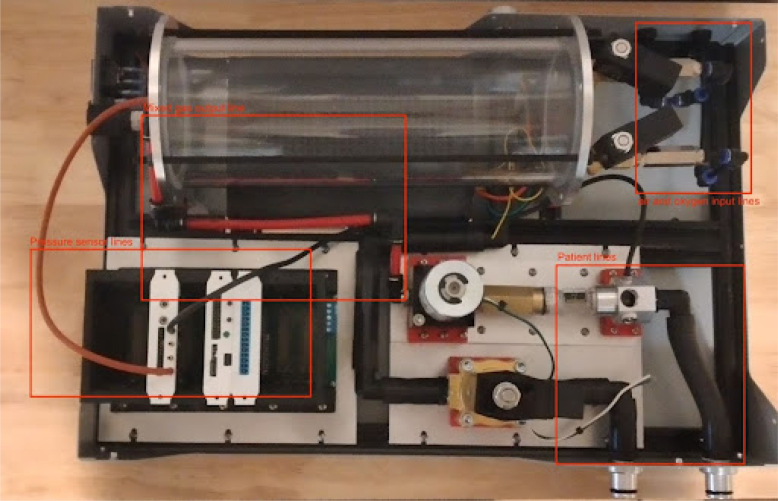
Patient exhalation diagram.

This is fairly straightforward, except for one connection. The 90 degree coupler in the Patient out to PEEP line has to be modified like Fig. 22. The final tube routing will look like Fig. 23.

• Make sure that your low-pressure sensing line goes to your low-pressure pressure sensor, and that your high-pressure sensing line goes to your high-pressure pressure sensor.
• Also make sure that you leave some slack in the sensing lines, so that they can be zip tied to the frame if needed.

**Control module power wiring**

On PolyVent machines, red is used for 24 V, black for GND, yellow for 5 V, and green for 3.3 V.

**Fig. 24:**
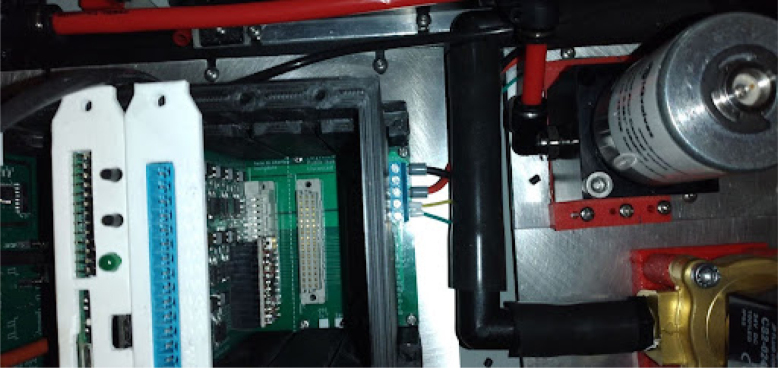
Control module wiring.

• If you used a different color code on your machine, you must account for it. The backplane has labels for GND, 24 V (PWR), 3.3 V, and 5 V.
• Once the wiring is done, slot an ESP32 card with the microcontroller into the backplane.
• Plug in the machine power, and flip the ON switch. If a green LED turns on in the ESP32 card, then the wiring was done correctly, and the rest of the cards can be added safely.
• The control module power wiring is depicted in Fig. 24.

**Valves board wiring**

**Fig. 25:**
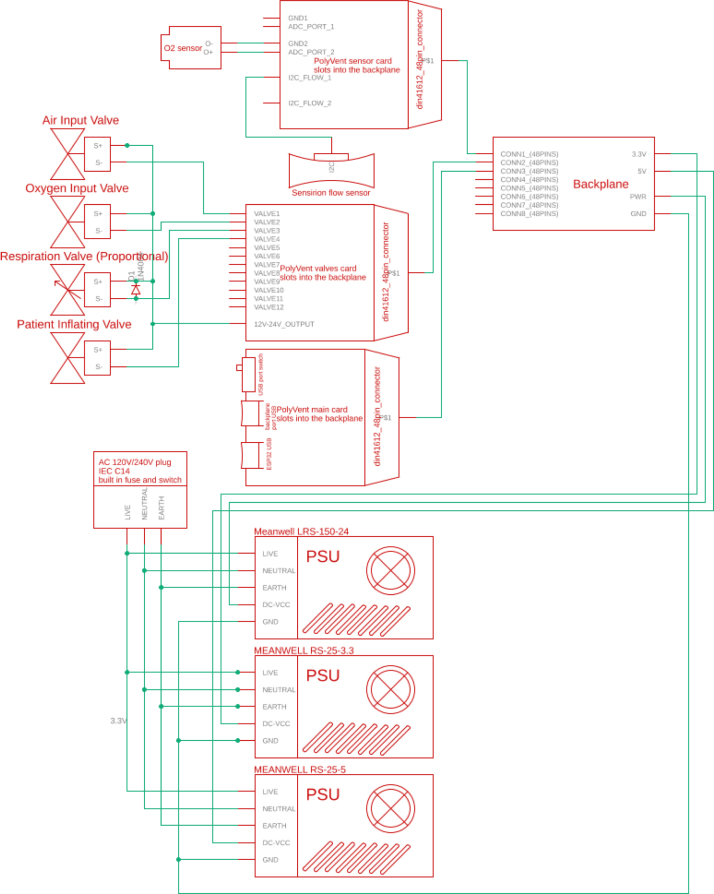
Control module wiring.

The valves can be wired according to the diagram in Fig. 25.

**Fig. 26:**
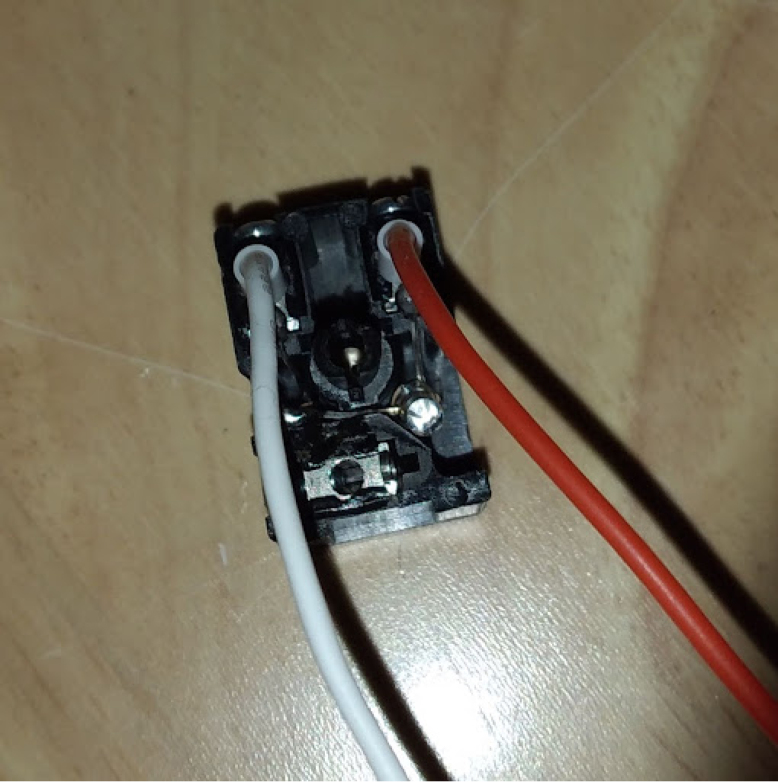
Valve wiring example.

**Fig. 27:**
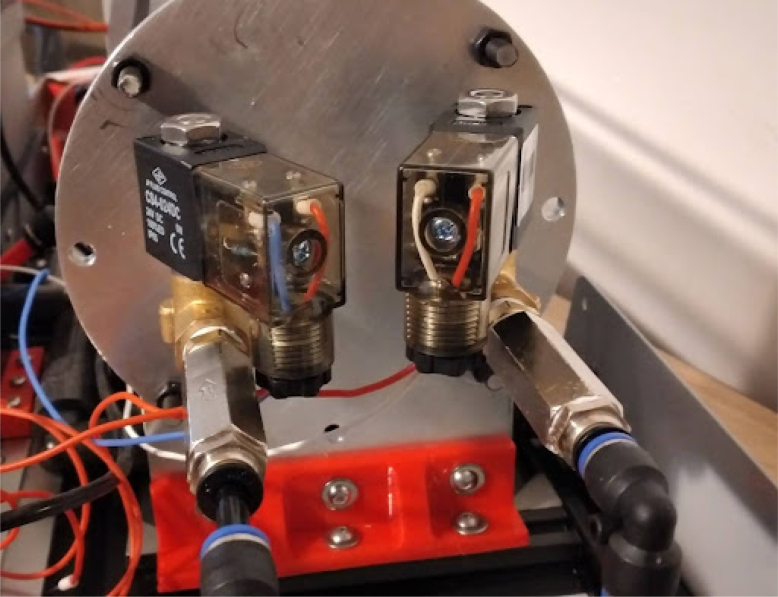
Valves installed on End Cap.

**Fig. 28:**
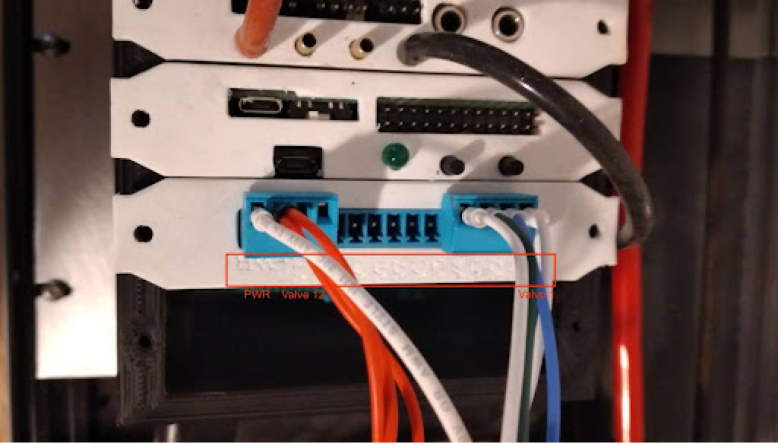
Installed expansion cards.

• Wires must be added to the DIN connectors of the valves, like in Fig. 26.
• Installed on the End Cap it looks like Fig. 27.
• The blue wire represents an oxygen valve, and the white one an air valve. They get wired into the installed valves card as shown in Fig. 28.

**O_2_ sensor installation and wiring**

The oxygen sensor in the PolyVent has an 18 month shelf life, so install only when it is ready for use.

**Fig. 29:**
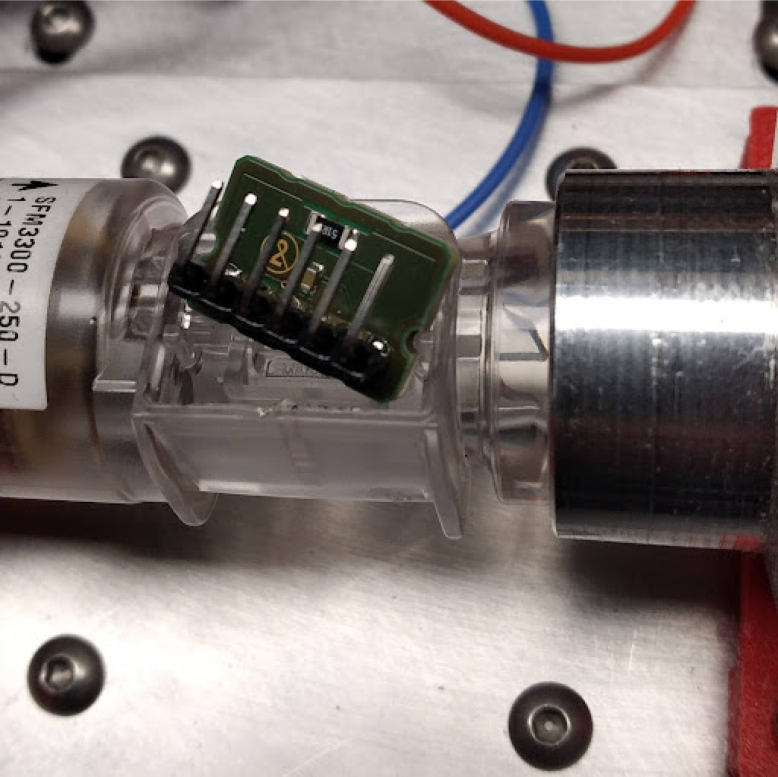
Un-epoxied Flow Sensor.

**Fig. 30:**
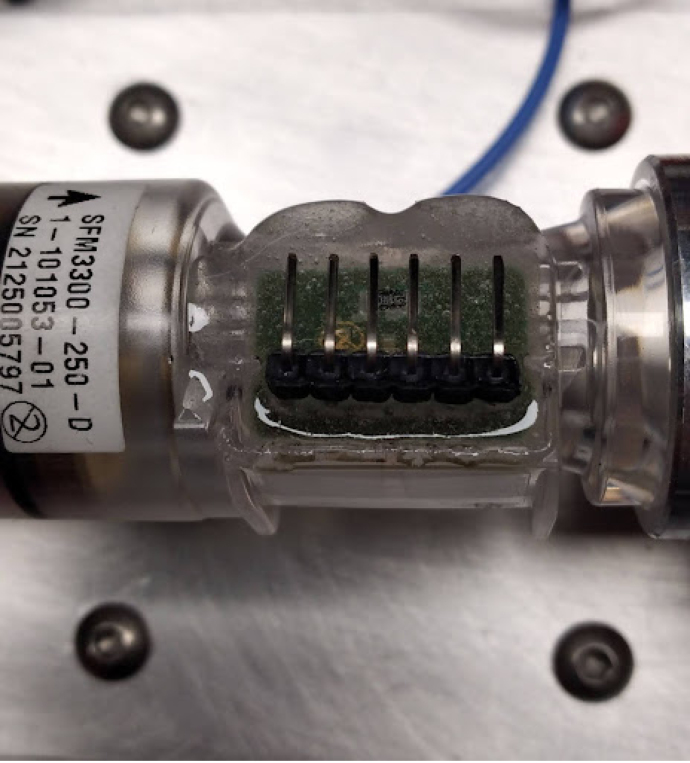
Epoxied flow sensor.

**Fig. 31:**
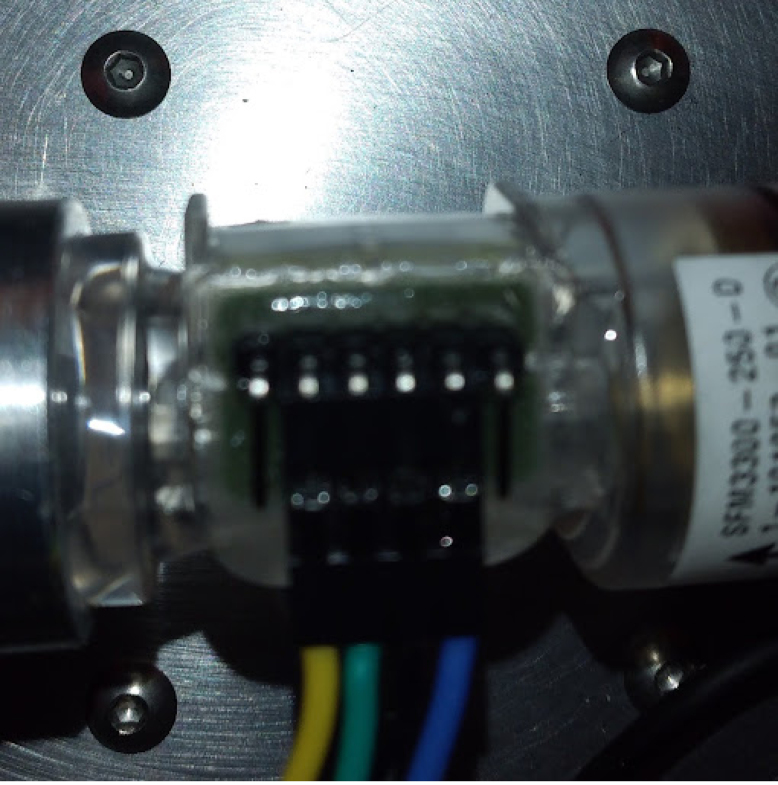
Mounted flow sensor.

**Fig. 32:**
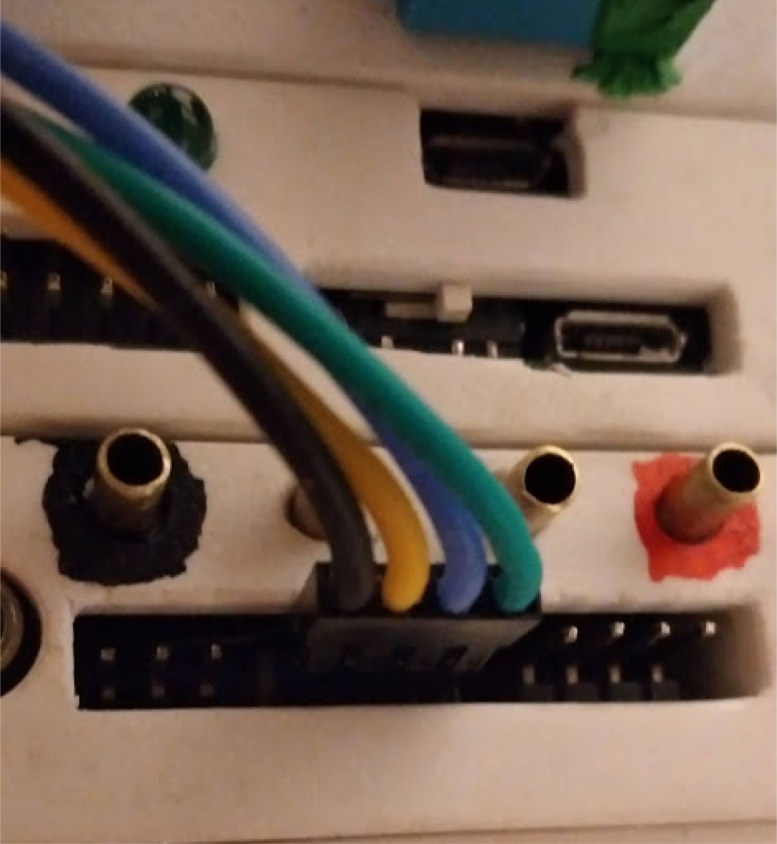
Flow sensor wiring.

**Fig. 33:**
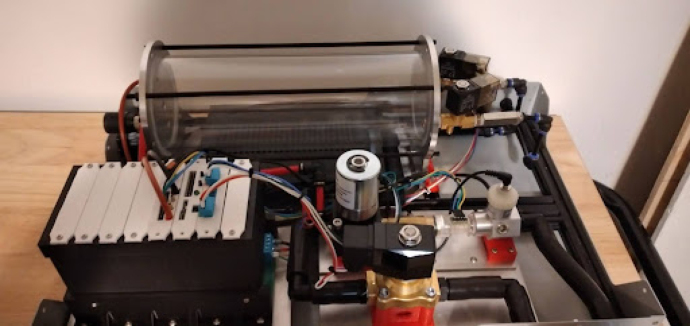
Full system.

• The oxygen sensor connects to the sensing card with a headphone jack. Mount it in the sensing manifold, in the top M16*1 mm thread.
• The flow sensor needs headers soldered to it. This is not ideal, but we have not been able to find clips for the sensor available anywhere. Sometimes, if too much heat is applied to the sensor during soldering, it comes off, like Fig. 29.
• This can be fixed with epoxy. The epoxied part looks like Fig. 30.
• The wiring can then be added as shown in Fig. 31, Fig. 32.
• Make sure to wire it this way, and notice that the ends of the connector have different pinouts. This is necessary for the flow sensor to work properly.

**Clean up the wiring and tubing**

• Zip tie the wiring to the frame, and label the connectors as needed.
• Add the blank covers to unused slots in the control module. The finalized wiring looks like Fig. 33.

**Finishing touches**

**Fig. 34:**
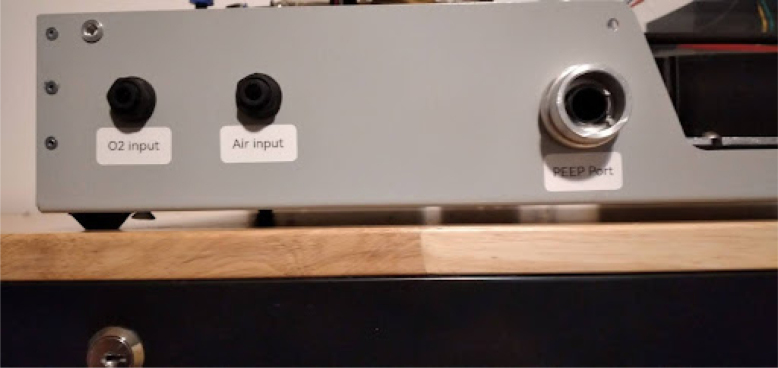
Labeled ports.

• Make sure the overpressure valve in the tank is set to 50 PSI.
• Add labels to the input and output couplers, Fig. 34.

**Form case top**

Note: The forming of the top has not yet been repeatably standardized. The current cover was formed by hand out of an acrylic sheet. A heat gun was used to gently heat and bend the sheet over a wooden form. This produced an attractive cover, but is not documentable. We are currently attempting to design a cold-formed polycarbonate cover with a repeatable design. For now, simply put on the case top.

**Fig. 35:**
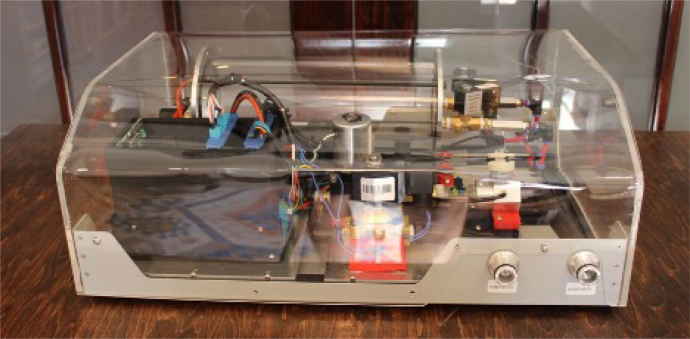
PolyVent with cover in place.

**The final product looks like**Fig. 35. **The Build/PolyVent is complete and ready for testing!**

**Starting the Machine without Pressure**

**Install VentOS**

Use a micro USB cable to load the most recent VentOS [14] software onto the ESP32. Connect it to the second micro USB slot on the control card (the one on the daughter board, not the main board). Follow the VentOS PlatformIO instructions for this. For example, you may issue the command:

**Figure:**
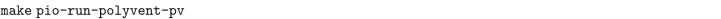

At this point, before installing the Valves Board itself in to the control module, you should be able to perform two tests:

• Power the system on and see if you can smell or see “smoke”. This is called a “smoke test”, and although a bit humorous, it is useful.
• Secondly, you should see startup messages and at least some debugging messages on the serial port, either with a terminal program, the Arduino IDE, or the PlatformIO.

You should be able to send commands via the serial port to the VentOS running the ESP32, and see acknowledgments of those commands. For some users, the easiest way to do this will be through the Arduino IDE [24], but a terminal application, such as PuTTY [25], should also work. Please see VentOS repo for documentation on this.

**Install the Valves Board Control Software**

• Use a micro USB cable to upload the Valves Board Code, which can be found here: https://gitlab.com/polyvent/polyvent_control_module/-/blob/master/Boards%20CAD/valves_card/firmware/valves_control_board_code/valves_control_board_code.ino.
• The easiest way to do this is with the Arduino IDE. Since the valves board microcontroller is a Sparkfun SAMD21 mini breakout, an extra boards library needs to be added to the Arduino IDE. Follow this tutorial to do this: https://learn.sparkfun.com/tutorials/installing-arduino-ide/board-add-ons-with-arduino-board-manager.
• In the boards manager, search SparkFun SAMD boards and install the library. To upload code to the valves card, you will then have to select Sparkfun SAMD21 mini breakout as your board.
• Once this board is plugged in and connected, you are ready to perform an additional test. When the PolyVent is powered on with the boards attached, you should be able to hear and feel the solenoids click about once every 10 s.
• Change the timing by sending the VentOS command to change the Breaths Per Minute (BPM) parameter. This format follows the PIRCS format [12].

**Starting the Machine with Pressure**

At this point you should be ready to apply a small amount of air pressure to the system.

• Check that the pop-off valve on the left side (downstream) port of the mixing chamber is set to 50 psi.
• We recommend you use a tank or compressor set to about 15 psi (less than the 50 psi it is rated for) for initial testing.
• It is easiest to test without pressurized oxygen at first by using pressured air in both the air and O_2_ inlets.
• With the system running but not attached to a test lung, slowly increase the input pressure.
• You should be able to feel a puff of air coming out of the “inspiration” port. The proportional valve may vibrate unpleasantly because the system is unable to produce pressure without a test lung attached.
• Then, shut down the machine and attach a breathing circuit and test lung [26].
• Now turn the machine back on and stand back, as a hose or test lung may pop loose.
• The system should pressurize the test lung, allowing air to escape out of the expiration port and the exhaust port in the back of the machine.
• If the machine is rhythmically filling the test lung and not overfilling it, you can begin experimenting with sending commands to change the PIP, breathing rate, and Inhalation to Exhalation ratio as defined by the VentOS documentation.

**WARNING:** If you observe the test lung overfilling, please make sure that the thin hose from the port of the pressure sensor on the valve card is properly connected. If this is not connected, the PolyVent cannot successfully regulate pressure and will overfill the test lung. Usually, a hose will pop loose, but the test lung itself could rupture in this situation.

**Fig. 36:**
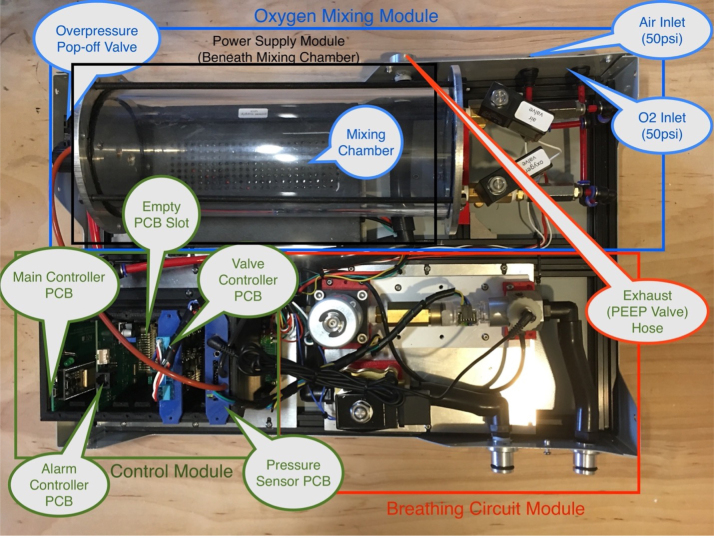
Overview of modules.

**Fig. 37:**
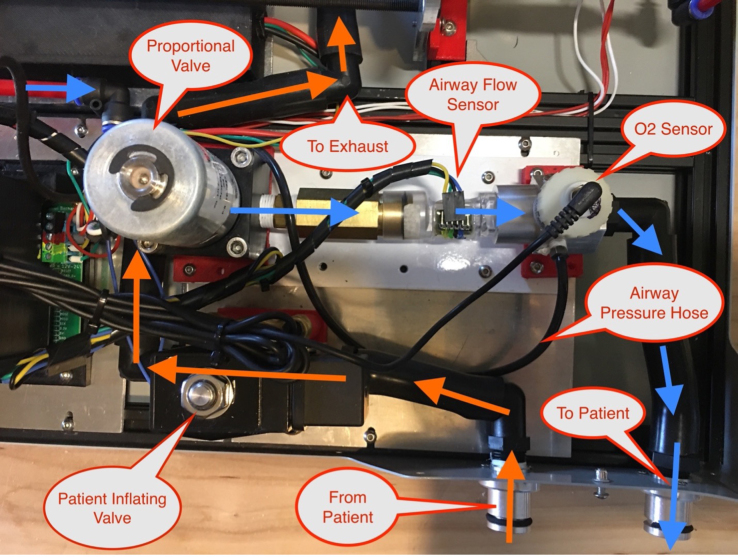
Airway detail.

## 6 Operation instructions

The PolyVent ventilates a lung system. In most cases, research scientists will use, or at least begin, with a test lung rather than a living or nonliving animal model. Simple plastic test lungs can adjust to represent different airway resistances and lung compliances. More sophisticated electronic test lungs could be used for researching patient-ventilator asynchrony (PVA).

**Fig. 38:**
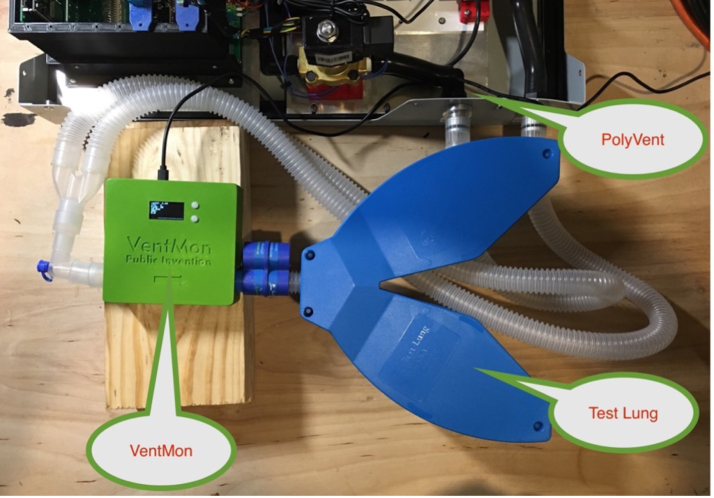
Test lung setup.

Fig. 38 depicts a typical experimental setup. This shows an electronic spirometer in a standard breath circuit tube connected to a blue plastic test lung. In this case, the spirometer is the VentMon open-source spirometer. It is an IoT enabled device that sends data to a public data cloud.

The VentDisplay software produces a real-time graphical display as shown in Fig. 41, Fig. 42. Since such traces are practically our only way to get visibility into pressure and flow in the airway under different conditions, this display is likely the end product of pulmonology research into PVA, new ventilation algorithms, new mechanical devices, etc. Fig. 41 depicts an explanation of the VentDisplay real-time data visualization.

The VentMon produces data in the Public Invention Respiration Data Standards (PIRDS) format [11]. This is a JSON data format that tags each sample with a millisecond timestamp. A researcher can use these log files to compute whatever they desire, or what is not apparent from the VentDisplay software. For example, the VentDisplay software does not currently allow visualization of more than three minutes of breath data at a time. However, a researcher in PVA likely desires a study of the breath data over an hours long period.

Commands transmitted over the serial port control the PolyVent’s behavior. This can be done manually using a terminal programmer or the Arduino IDE. These commands are expressed in a JSON data format called the Public Invention Respiration Control Standard (PIRCS) [12]. Both PIRCS and PIRDS are open formats intended to be universal and reused by other respiration technology.

The PIRCS format uses a JSON binding that allows the user to set clinical parameters. Although clinical pulmonology is evolving, there are essentially a fixed set of parameters common to all ventilation, which can be expressed in the PIRCS format, and change the PolyVent behavior:

• M : Mode (Pressure Control Ventilation, Volume Control Ventilation, etc.)
• P : Target Pressure
• B : Breaths per minute times 10
• I : E:I Exhalation to Inhalation time Ratio
• O : Oxygen FiO_2_,
• S : Emergency Stop

The PIRCS format allows a user to set other parameters which are not yet implemented by the PolyVent. The FiO_2_ control of mixing has been tested with nitrogen and oxygen.

For example, to change the number of breaths per minute to 12, enter:

**Figure:**
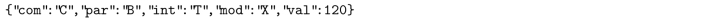

**An Overview of the Software Operation**

For complete information, please see the VentOS documentation. In a nutshell, the VentOS runs a “superloop” or “simple loop” architecture with an explicit task manager.

One of the VentOS’s tasks is to listen on the serial port for commands. The other task is to control the machine itself. A second board controls the solenoid valves, which is communicated with via SPI communication in the backplane of the control module.

The main control loop reads the pressure and adjusts the proportional solenoid valve to be slightly more open or slightly more closed to maintain a steady target pressure. A PID controller internally controls this function. When the inhalation phase is over, the PIV valve opens.

VentOS has considerable internal debugging features, but only an experienced C++/Arduino programmer can use them. If you become stuck, please contact Public Invention for free technical support.

Although considerable work has been put into a universal GUI [27], it is not yet functional at the time of this writing.

**Troubleshooting and Common Problems**

The PolyVent is relatively easy to troubleshoot. A classroom exercise of finding various, intentionally introduced problems was performed as a 3-hour educational module [28].

Generally speaking, understanding the air circuit described in Fig. 37 allows one to diagnose a physical problem. Additionally, anyone with experience using Arduino embedded systems can easily debug software problems. Occasionally, however, when using very high pressure in the course of testing, the valve sensing hose will pop off.

## 7 Validation and characterization

The PolyVent has been used for classroom instruction, including exercises such as total recreation of the hardware and significant variation of the clinical parameters.

**PolyVent Classroom Rebuilds**

**Fig. 39:**
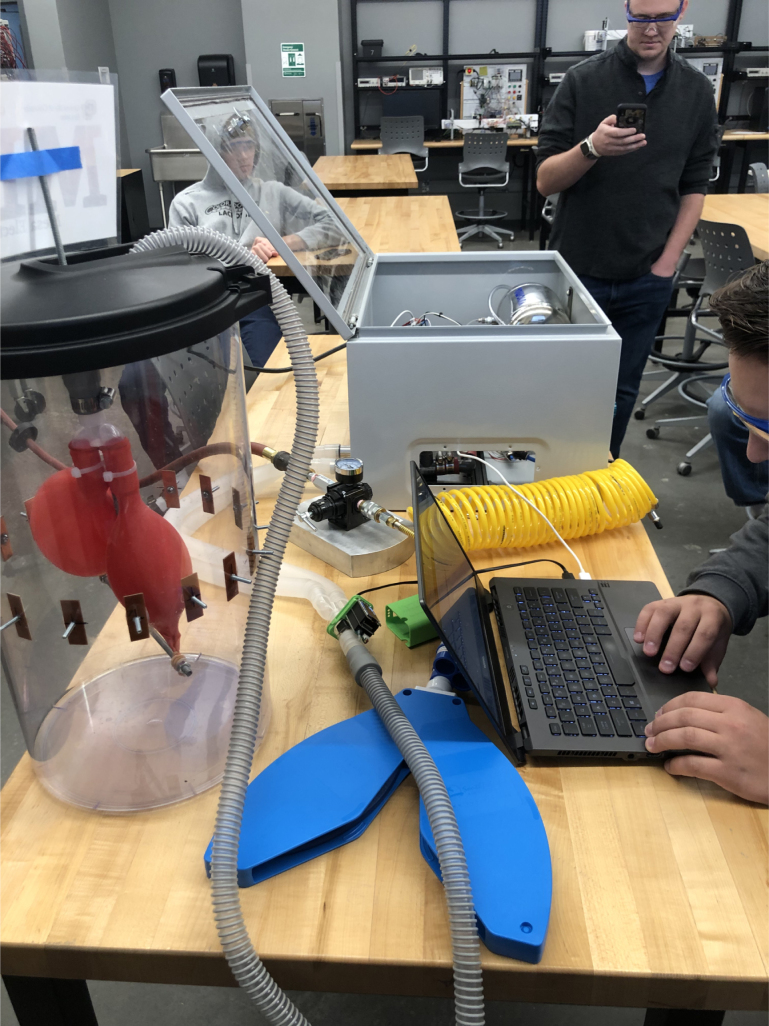
Colorado Mesa University Students Operating their PolyVent.

A team of eight senior mechanical engineering students in the University of Colorado Boulder/Colorado Mesa University Partnership Program (Grand Junction, CO) successfully recreated the PolyVent as part of an elective course in Bioengineering in November of 2024 (Fig. 39). For all the students in the class, it was their first exposure to open-source design. Students were divided into teams each dedicated to the major areas of the PolyVent: an enclosure team, a mixing chamber team, a breathing circuit team, and team which oversaw the electronics. Students were supported by the instructor as well as the PolyVent inventors. All valves, sensors, and other electronics were implemented in an identical manner to the PolyVent.

**Fig. 40:**
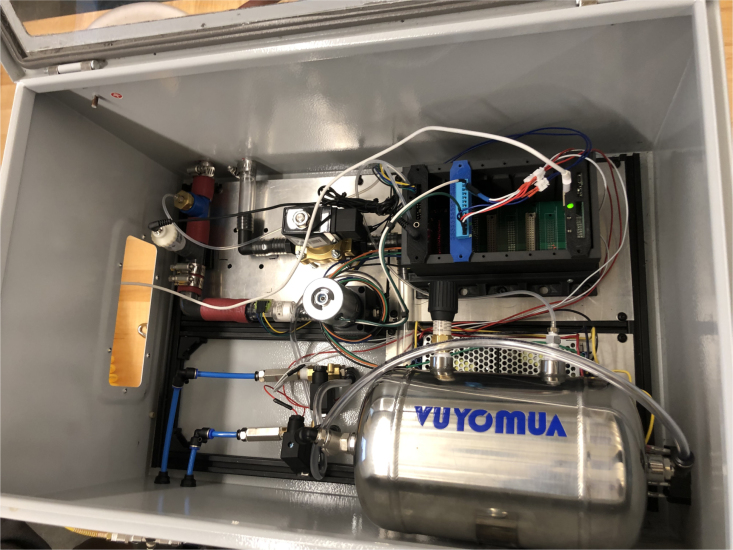
Colorado Mesa University PolyVent.

Small modifications were made to the other designs aspects to expedite the building process. For example, an electrical panel box was used as the enclosure instead of fabricating one from scratch. Students also added acrylic windows to the box, which allowed others to readily see inside. Students working on the breathing circuit took parts to a local hardware store in order utilize as many readily available connectors as possible, and they successfully found a substitution for the machined component that holds the oxygen sensor. See Fig. 40.

Instead of writing reports, students were asked to generate corresponding files and instructions which will be added to a publicly available repository for the project. This class project was incorporated as part of a module on pulmonary biomechanics and mechanical ventilation. It will be built upon in future years, as the instructor of the course aims to modify the ventilator for research purposes.

**PolyVent in Classroom Research**

**Fig. 41:**
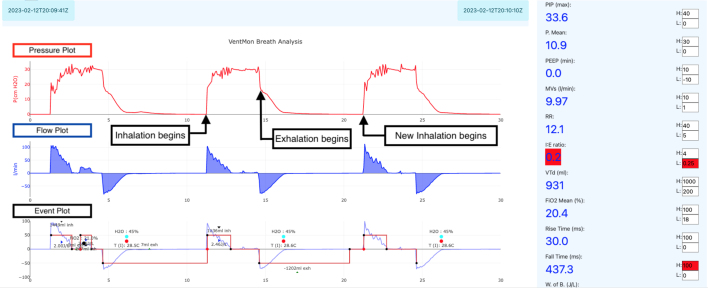
VentDisplay explanation.

Inspection of the spirometry visual traces, as shown in Fig. 41, validates its function throughout typical clinical parameter ranges.

**Fig. 42:**
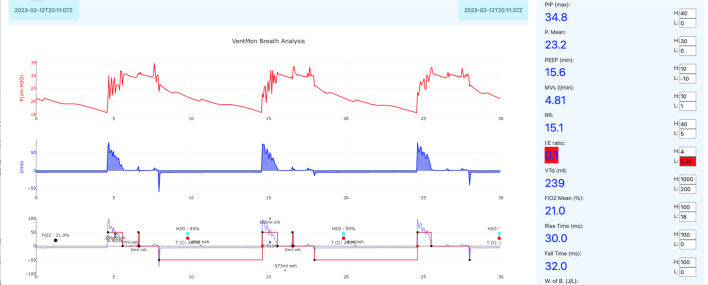
Breath trace of obstructed exhalation path.

Fig. 42 is a breath trace of an intentionally obstructed exhalation airway. In this case, a ball bearing was placed in the airway, partially obstructing it and preventing rapid flow. This is an actual exercise presented to students in the classroom. The students were asked to diagnose the problem from a visual inspection of the breath traces. As shown, the exhalation flow is very slow and spread out over a long period of time when compared to an unobstructed airway such as Fig. 41.

The PolyVent is a functional open source research tool, but is not currently a medical device intended for use on patients. If a firm were to build a medical ventilator using the PolyVent design as a starting point, they would start with a tested functioning design, but would run into a number of limitations and unimplemented features:

• The PolyVent has been tested in a high-oxygen environment, but only briefly.
• A clinical User Interface (with physical knobs or a touch screen) has not yet been implemented.
• Volume Control Ventilation Mode has not yet been implemented.
• A multi-week endurance test has not yet been done.
• The PolyVent hardware was tested with the General Purpose Alarm Device hardware to make flashing lights and loud sirens to call attention to a problem, but the VentOS software has not yet been programmed to detect and activate alarms.
• The manufacture of the cover remains unstandardized and requires skilled manual labor.
• The PolyVent pressure vessel has not been designed to the specifications in the ASME Boiler and Pressure Vessel Code. Furthermore, microfractures have been found in the acrylic tank of the PolyVent’s pressure vessel. The authors recommend that an off-the-shelf pressure vessel be used until this is investigated further.

The PolyVent, in combination with the VentMon spirometer, VentDisplay dynamical real-time website, and VentOS software, is a molecule or constellation of interacting components, each of which is free and modifiable. A medical ventilator typically attempts to pack all of the components into a single compact case. The PolyVent, like open-source software in general, assumes that it is better and more flexible to have modular components which are independently reusable. The PolyVent machine can be considered the medical gas production module in such a functional system of modules. It is in this sense an ideal tool for researchers who value openness in licensing, design, and philosophy.

## CRediT authorship contribution statement

**Robert L. Read:** Writing – review & editing, Writing – original draft, Supervision, Software, Resources, Project administration, Methodology, Investigation, Funding acquisition. **Nathaniel Bechard:** Writing – original draft, Visualization, Validation, Project administration, Methodology, Investigation, Conceptualization. **Victor Suturin:** Supervision, Project administration, Methodology, Investigation, Formal analysis, Conceptualization. **Antal Zuiderwijk:** Writing – review & editing, Methodology, Investigation. **Michelle Mellenthin:** Writing – review & editing, Validation.

## Ethics statements

Not relevant.

## Declaration of competing interest

The authors declare that they have no known competing financial interests or personal relationships that could have appeared to influence the work reported in this paper.
